# Supplementary material for: Anxiety classification in virtual reality using biosensors: A mini scoping review
Source: PLoS One. 2023 Jul 10;18(7):e0287984. doi: 10.1371/journal.pone.0287984 (PMC10332625; doi:10.1371/journal.pone.0287984)
Supplement: S1 File — (PDF) [file pone.0287984.s002.pdf]

Supplementary Material

Quality Assessment Scale

- QS1:Was there a clear statement of the aims of the research?
  - Yes: Findings are explicitly stated and adequately discussed in relation to the initial research question
  - No: No information
  - Partially: The aims are stated but not sufficiently discussed
- QS2: Were the methods used justified in the paper?
  - Yes: The biosignals and machine learning methods used were described with the reasons and it had face validity
  - No: Not explained
  - Partially: Methods were explained but either not justified or not detailed enough and/or were open for interpretation
- QS3: Was the recruitment strategy appropriate to the aims of the research?
  - Yes: Sample size and population were determined before the research and justified as to why this population is the best for the aims of the study
  - No: There is no information regarding how the participants were selected
  - Partially: There was some information on the recruitment process but eligibility criteria
- QS4: Have ethical issues been taken into consideration?
  - Yes: The study received ethical approval from a committee and clearly states the policy number
  - No: No information
  - Partially: Ethical concerns were explained clearly for the reader to make out that enough was done to avoid them, but there was no mention of ethical approval from a committee

- QS5: Were the classification model(s) used clearly explained and sufficient?
  - Yes: There was a clear explanation of the analysis done and it was appropriate to study, specifically with diagrams and justification for them being used
  - No: No information
  - Partially: Process was briefly mentioned but without sufficient detail and/or open to interpretation
- QS6: Is there a clear statement of findings?
  - Yes: Findings are explicitly stated and adequately discussed in relation to the initial research question
  - No: No information
  - Partially: The findings are stated but not adequately discussed
- QS7: Are the limitations of the study thoroughly discussed?
  - Yes: There are references to sample and technical reasons that the study might not be generalisable
  - No: No limitations discussed
  - Partially: There are some limitations discussed, but there are some concerns that were not mentioned
- QS8: Are the contributions of the study to the literature and future research discussed?
  - Yes: There is an adequate discussion about the contribution of the study
  - No: No mention
  - Partially: Contributions to the literature are superficially discussed, with little evidence or justification from the findings of the paper and where they fit in the area
