# Supplementary material for: Anxiety classification in virtual reality using biosensors: A mini scoping review
Source: PLoS One. 2023 Jul 10;18(7):e0287984. doi: 10.1371/journal.pone.0287984 (PMC10332625; doi:10.1371/journal.pone.0287984)
Supplement: S3 File — (DOCX) [file pone.0287984.s004.docx]

- Reference (First author / Year / Journal citation)
- Sample size:
- Type(s) of physiological/behavioural infomation collected and used:
- Anxiety measurement/inducing method(s) used:
- Number of outputs for classification:
- Quality score:
- Highest Accuracy Achieved:
- Notes (and additional bibliography):
