# Supplementary material for: Anxiety classification in virtual reality using biosensors: A mini scoping review
Source: PLoS One. 2023 Jul 10;18(7):e0287984. doi: 10.1371/journal.pone.0287984 (PMC10332625; doi:10.1371/journal.pone.0287984)
Supplement: Attachment — Submitted filename: Response to Reviewers .pdf [file pone.0287984.s007.pdf]

RESPONSE TO REVIEWERS

Reviewer 1

We thank the reviewer for their valuable comments. We tried to address the issues raised in the following steps:

> The authors have not provided sufficient details of about the anxiety, VR and biosensors in the introduction part. They should expand their introduction providing more explanation of these terms which makes the basis of their work. Building on the knowledge of anxiety (or stress), VR and Biosensors, the importance of anxiety classification should be presented. The authors describes very little about its applications. More applications should be provided as evidence and their limitations should be included in the review.

As per this suggestion, we have expanded upon our introduction, adding the following sections:

- Definition of Anxiety
- Stress Response
- Physiological and Behavioural Measures

We have also added some extra paragraphs into each section, including application areas.

> The authors tried to establish their research questions but reading ahead it seems that they don't answered their RQ and the review doesn't look like a systemic approach. They should stick to their own RQs and provide detail answers in the light of published literature.

We have reevaluated the research questions to address this problem, as well as changed the methods into a scoping review as per another reviewer's suggestion. Due to the change from systematic review to scoping review, we have come up with some changes to better investigate the literature.

> Inlusion/exculsion criteria should be presented in a simple table.

Agreed, added Table 1.

> No details of the classification's methods are provided. Mentioning only the name classifiers does not provide any useful information to the researchers in the field.

We have expanded upon this section including some more information such as data preparation, feature extraction and selection, time windows and validation methods. Due to different types of measures, there is a lot of heterogeneity in this area and the information was kept brief to provide insight into current methods in the literature.

> The discussion is just a summery of what they have presented earlier and not a real discussion over the problem of topic.

We have improved upon the discussion and added the following subsections:

- Limitations
- Future directions

We believe that these changes have improved the quality of our paper significantly. We are happy to make further adjustments if you have any other concerns.

Reviewer 2

Thank you very much for all your valuable insight, especially into PRISMA and systematic reviews. We tried to address your suggestions in the following steps:

> 1.In general, systematic reviews (SR) are intended to summarize knowledge on a particular topic, to provide specific practical guidelines for clinicians, and recommendations for future research. It is important that such conclusions can be drawn in relation to a specific population.

Thank you very much for your comment. We agree that a systematic review of the topic may not be the most suitable, especially considering how new it is. We have changed the submission into a scoping review.

> 2. The research question is usually very precise and allows to answer a specific clinical problem. Most of the times, a systematic review decides on the effectiveness of a therapy or compares different approaches to therapy. Meanwhile, the research questions posed by the Authors are precise; there are many of them, but they do not resolve the clinical problem.

We have changed the review type to scoping and adjusted the research questions accordingly.

> 3. One of the most important elements of SR is the creation of the PICO and the inclusion and exclusion criteria. These elements are intended to ensure precision throughout the literature review process.

We have avoided using PICO because it is difficult to apply to computer science studies. We believe that changing the review type has made our questions more relevant. We also changed the inclusion and exclusion criteria into a table for enhanced comprehension.

> 4. SR requires a very precise approach. The guidelines clearly show the need to register the protocol a priori, or the participation of two independent Reviewers during the screening of abstracts, screening of full texts, and the qualitative assessment of the included articles. All these assumptions are intended to increase the relevance, reliability and objectivity of the review.

We agree with the reviewer on all accounts. The current submission is a mini-review, intended to inform on a small area of research, evaluate current progress and identify gaps in the literature. We do not believe a single reviewer would be enough for a detailed clinical systematic review or

a meta-analysis. However, we do believe that it is useful for a quick scan of the literature to inform future work.

> In conclusion, the Reviewer greatly appreciates the effort put into the review by the authors. Following the steps of the CONSORT guidelines is very engaging and demanding. The recommendations are as follows:
> a) Change the name of the study design to “literature review” or “scoping review”; however, the Reviewer believes that "scoping review" would be a better option, as the study is based on many research questions and has an exploratory nature.

Agreed and changed.

> b) It is recommended to delete the PRISMA checklist, the Authors should replace it with the PRISMA-ScR checklist.

Agreed, removed previous PRISMA and added File 2, PRISMA-ScR checklist.

Thank you very much for your insight on systematic and scoping reviews. Structured reviews are less common in computer science than in health, and we are very grateful to have an expert weigh in on the appropriate methods. We believe that the study is a lot more appropriately structured and labelled as a scoping review. We are grateful for your comments.

Reviewer 3

> The authors mentioned in the beginning in the article that these two terms will be used interchangeably. However, this is not supported by the psychology literature, which clearly differentiates these terms. The author is pointed to the DSM 5 for detailed operationalizations of these terms.

We agree that the wording of this section gives the wrong impression. We have not intended to argue that stress and anxiety are the same concept. What we tried to convey was that despite there being a clear difference that needs to be acknowledged, the methods used for detecting each of these states share very similar traits, therefore it is useful to refer to them both during our review. We added a section called "definition of anxiety", where we discuss this a bit more.

> Citations were relevant, with most being from the past 10 years. However, there were still some citations that seemed to be out-of-date. Unless these are seminal articles, it is recommended that the author update these citations.

We have added more recent citations into the introduction where the previous citations were outdated. Most of these citations are related to the use of VR in psychotherapy.

> This table results answer the RQs; however, in the results section overall, it would be better to have a separate section based on each RQ because it was difficult to locate whether or not all the RQs were properly answered. Again, labeling sections using RQs is recommended.

We have reorganised the results section to better account for each question. Note that the questions have also been changed to better reflect the study more appropriately being titled a scoping review based on the suggestion of another reviewer.

> There were significant limitations in the study starting from finding a definition of anxiety itself as different studies shows different definitions. However, the author is again referred to the DSM 5 for a standardized operationalization of the construct.

Thank you for this valuable suggestion. Hopefully, we addressed these issues a little better and discussed them more clearly.

We believe that your suggestions have led to the betterment of our manuscript. We are very grateful for your time and suggestions.

Journal Requirements

Thank you for the information.

- The file names have been corrected appropriately.
- The funding information has been removed from the manuscript.
