# Supplementary material for: Anxiety classification in virtual reality using biosensors: A mini scoping review
Source: PLoS One. 2023 Jul 10;18(7):e0287984. doi: 10.1371/journal.pone.0287984 (PMC10332625; doi:10.1371/journal.pone.0287984)
Supplement: Attachment — Submitted filename: Revision.docx [file pone.0287984.s005.docx]

The Authors present a review on anxiety classification in virtual reality scenarios. Virtual reality has become a very widely used technology in many areas, including healthcare or training in dangerous professions. Research shows many advantages of this solution and emphasizes its effectiveness. The limitations of this technology should also be noted. Researchers point out several significant side effects of using virtual reality. One of them is stress and anxiety, which can be the result of long-term exposure to a stressor. It is known that the measurement of these symptoms is not simple, especially in real time when exposed to intervention in a virtual environment. Therefore, the Reviewer would like to thank the Authors for conducting such interesting secondary research. This article is quite well-prepared and conveys important knowledge; however, there are some issues that need to be revised.

**GENERAL STRENGTHS**

My knowledge in the field of biosensors is limited; however, because of my interest in modern technologies (including virtual reality), I consider this research to be very important in the context of the development of this technology. Therefore, I believe that the article presents important aspects related to the evaluation of side effects of therapy in a virtual environment. This is especially relevant in my field of clinical research. Content-wise, I have no complaints.

**GENERAL WEAKNESSES**

My expertise in the field of research design allows me to draw attention to one aspect. I recommend the Authors to change the study design. From my point of view, this study cannot be called a "systematic review". Please find below the most important comments listed point by point that explain my recommendation.

1. In general, systematic reviews (SR) are intended to summarize knowledge on a particular topic, to provide specific practical guidelines for clinicians, and recommendations for future research. It is important that such conclusions can be drawn in relation to a specific population.

2. The research question is usually very precise and allows to answer a specific clinical problem. Most of the times, a systematic review decides on the effectiveness of a therapy or compares different approaches to therapy. Meanwhile, the research questions posed by the Authors are precise; there are many of them, but they do not resolve the clinical problem.

3. One of the most important elements of SR is the creation of the PICO and the inclusion and exclusion criteria. These elements are intended to ensure precision throughout the literature review process.

4. SR requires a very precise approach. The guidelines clearly show the need to register the protocol a priori, or the participation of two independent Reviewers during the screening of abstracts, screening of full texts, and the qualitative assessment of the included articles. All these assumptions are intended to increase the relevance, reliability and objectivity of the review.

In conclusion, the Reviewer greatly appreciates the effort put into the review by the authors. Following the steps of the CONSORT guidelines is very engaging and demanding. The recommendations are as follows:

a) Change the name of the study design to “literature review” or “scoping review”; however, the Reviewer believes that "scoping review" would be a better option, as the study is based on many research questions and has an exploratory nature.

b) It is recommended to delete the PRISMA checklist, the Authors should replace it with the PRISMA-ScR checklist.

**MINOR CONCERNS**

1. Please consider presenting the results of the qualitative assessment in a graphical form, e.g., in a form similar to the RoB-2 figure.

2. Please try to relate the results to the qualitative assessment of the included articles.

3. In the discussion, please separate the sub-sections "Limitations" and "Recommendations for further research”. These are the areas from which the most can be drawn when designing future research. Therefore, they should be clearly highlighted.

4. In Figure 1, please indicate the number of records found in each database:

Records identified from:

Databases (n = 1749):

- IEEE Xplore (n = )
- ACM Digital Library (n = )
- Scopus (n = )
- Web of Science (n = )

5. Please present “Data extraction form” in the form of a table.

Yours sincerely,

Reviewer
