# Supplementary material for: Anxiety classification in virtual reality using biosensors: A mini scoping review
Source: PLoS One. 2023 Jul 10;18(7):e0287984. doi: 10.1371/journal.pone.0287984 (PMC10332625; doi:10.1371/journal.pone.0287984)
Supplement: Attachment — Submitted filename: Review for Mini Systematic Review.docx [file pone.0287984.s006.docx]

**Anxiety Classification in Virtual Reality Using Biosensors: A Mini Systematic Review**

**Abstract**

In the abstract section, research purpose, methods, and findings and conclusions of the review was written in 200–300 words in a concise manner.

**Background of review and research question**

The introduction section clearly stated the research problem and the purpose of this study. There were a total of five research questions that were stated in a straightforward fashion.

Research problem has been stated that anxiety classification in VR is still a challenge, however, there was no citation that backs up that specific statement. The article introduces a gap to investigate objective measures using physiological and behavioral information in VR.

The research purpose was stated clearly that it is to assess the evidence on whether anxiety can be accurately classified in VR.

The background provided enough context for the readers to understand what this study is going to focus on. There were a couple of constructs that were mentioned such as stress and anxiety. And the authors mentioned in the beginning in the article that these two terms will be used interchangeably. However, this is not supported by the psychology literature, which clearly differentiates these terms. The author is pointed to the DSM 5 for detailed operationalizations of these terms. Citations were relevant, with most being from the past 10 years. However, there were still some citations that seemed to be out-of-date. Unless these are seminal articles, it is recommended that the author update these citations.

The authors has provided sufficient detail on how to predict anxiety using psychophysiological measures such heart rate, blood pressure, skin conductivity, behavioral measures such as pupil dilation and breathing. This gives an overview to a novice reader on what psychophysiological measures are in general. The author points to prior studies used to predict levels of anxiety using machine learning models; however, in this current study the author stated clearly that it is using biosensors in classifying anxiety in VR.

**Methods**

The criteria for inclusion and exclusion of studies and any quality criteria applied was well described in the materials and methods section in bullet points which was easy to read for the readers. The search string for databases were well described as well.

The authors stated that the review was not registered, and a protocol was not prepared. However, it is helpful that the authors provided a copy of the PRISMA chart for the readers to see the process of the data collection and also the data extraction form.

**Findings of the review/Discussion**

In this section, reasons for exclusion of potentially relevant studies was clearly indicated in the first 2 paragraphs in the results section.

The authors stated in the “Physiological and behavioral measures” section that there was lack of information on how a variety of biosensors/measures are being used which shows that there is gap in studies.

In the 3rd paragraph under “Physiological and behavioral measures” section, the author seems to answer RQ1 on challenges for accurately assessing anxiety in VR in real time. Labeling the headings such that the reader is able to clearly identify which research question is being addressed is recommended.

In Table 1, the findings were well presented for the readers to review. This table results answer the RQs; however, in the results section overall, it would be better to have a separate section based on each RQ because it was difficult to locate whether or not all the RQs were properly answered. Again, labeling sections using RQs is recommended.

**Conclusions**

There were significant limitations in the study starting from finding a definition of anxiety itself as different studies shows different definitions. However, the author is again referred to the DSM 5 for a standardized operationalization of the construct. The authors stated a variety of reasons for the limitations of this mini systematic review in the discussion section. Future directions were clearly stated in the manuscript for the readers.
